# Correlating measurements across samples improves accuracy of large-scale expression profile experiments

**DOI:** 10.1186/gb-2009-10-12-r143

**Published:** 2009-12-30

**Authors:** Mariano Javier Alvarez, Pavel Sumazin, Presha Rajbhandari, Andrea Califano

**Affiliations:** 1 Joint Centers for Systems Biology, Columbia University, 2960 Broadway, New York, NY 10027-6900, USA; 2 Department of Biomedical Informatics and Institute for Cancer Genetics and Herbert Irving Comprehensive Cancer Center, Columbia University, 2960 Broadway, New York, NY 10027-6900, USA

## Abstract

Gene expression profiling technologies suffer from poor reproducibility across replicate experiments. However, when analyzing large datasets, probe-level expression profile correlation can help identify flawed probes and lead to the construction of truer probe sets with improved reproducibility. We describe methods to eliminate uninformative and flawed probes, account for dependence between probes, and address variability due to transcript-isoform mixtures. We test and validate our approach on Affymetrix microarrays and outline their future adaptation to other technologies.

## Abstract 2

Cleaner is a method for removing uninformative and flawed probes from microarray experiment data, thus improving reproducibility between replicate experiments.

## Background

Gene expression profiling is a valuable technique for studying cell phenotype at the molecular level. Microarray gene expression profiling, in particular, is unquestionably the most widely adopted molecular profiling technique, used virtually throughout the life-sciences, and deep-sequencing based approaches are slated to further improve our ability to monitor gene transcripts in the cell. However, since the inception of the technology, the accuracy of gene expression profiles has been questioned due to relatively poor reproducibility [1-3]. Numerous studies have attempted to improve accuracy and reproducibility by applying filtering methods and improving data processing and normalization [4-7], but both technology-specific and technology-independent aspects of the gathering and analysis of this data modality remain challenging. For instance, a recently addressed gene expression profile-specific challenge is posed by probe designs that become rapidly outdated due to changes in genomic sequences and their annotations, and probe-set remapping using up-to-date genomic annotation has been repeatedly shown to improve Affymetrix expression microarray accuracy [8,9]. To improve reader comprehension, we note that in this correspondence we address microarray expression profiling technical challenges at the probe level and we are careful to distinguish between individual probes (that is, 25-mer oligonucleotide sequences), Affymetrix probe sets (sets of 25-mer probes designed to span a target region based on a UniGene cluster), and our own probe clusters. Technology-independent challenges that are at best only partially resolved are related to tissue-specific transcript isoforms, post-transcriptional modifications, and polymorphisms that can affect measurement accuracy in a context-specific fashion. Because of their complex and poorly understood nature, accounting for all of these features individually is a prohibitive task, and technological advancements alone are not likely to resolve them.

We noticed that prior efforts to address these challenges were mostly focused on improving accuracy for standalone expression measurements, and largely disregarded the increasing availability of gene-expression profiles representing a diversity of phenotypic or molecular contexts for the same cellular system [10,11]. This diversity is quite valuable, as it allows for monitoring transcript isoforms and increasing measurement accuracy by assembling transcript-specific clusters of correlated probes across large and diverse sample sets. Statistical methods that take advantage of this diversity of expression measurements can identify and even correct biases that typically result in measurement inaccuracies. We reasoned that if distinct probes monitor the same transcript isoform, then their measurements should be highly correlated across large gene expression profile datasets. This simple idea, which has not been previously used for the identification of informative probes, can be used to substantially improve expression measurement accuracy and to monitor alternative splice variants in the cell. Our proposed algorithm, Cleaner, implements this idea for Affymetrix GeneChip microarrays and may be extended in a straightforward fashion to other technologies.

**Figure 1:**
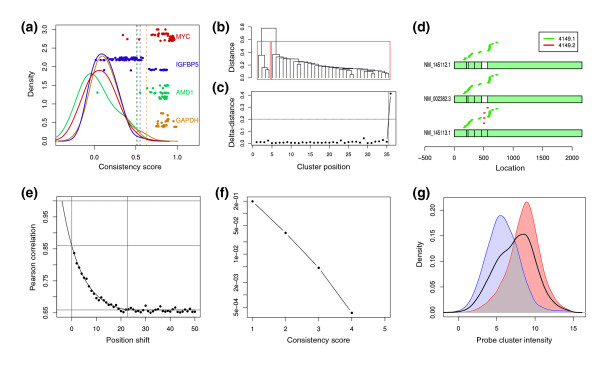
Cleaner on 152 B-cell samples profiled on U95Av2 chips. (a) Probe consistency scores for four genes (distinguished by color) and their corresponding null density distributions. Dots represent probes and are plotted according to consistency scores (horizontal axis) and distances from the transcript end (vertical axis). Solid lines depict null density distributions and dotted vertical lines are drawn at their 99 percentile. Consistent probes are to the right of their respective dotted lines. (b) Unsupervised hierarchical clustering of probes mapping to MAX (all isoforms); naturally occurring probe clusters are highlight in red. (c) Relative distance between each consecutive cluster in the dendrogram in (b); the right-most point represents the distance between highlighted clusters in (b). (d) Three known isoforms for MAX and the mapping positions of probes belonging to the two Cleaner probe clusters for MAX; probes from probe cluster 4149.2 are mapped to the splice-variant fifth exon, and probes from 4149.1 measure a convolution of the two transcripts. (e) The mean of Pearson correlations between overlapping and neighboring probes depends on the distance between them, and it is closely modeled by an exponential function. (f) False discovery rate (FDR) as a function of the consistency score, as estimated by permutation testing; no probe-cluster with consistency scores higher than 4 were identified in permuted clusters. (g) Probe-cluster consistency scores are correlated to their MAS5-assigned intensity, as measured before pruning. However, the intersection between distributions for the 4,702 consistent probe clusters (red), 3,708 inconsistent probe clusters (blue), and all probe clusters (black line) suggests that probe-cluster intensity does not perfectly predict probe-cluster consistency.

To illustrate the algorithm, without loss of generality, we focus on remapping, filtering and clustering probes in Affymetrix GeneChip microarrays, one of the most popular genome-wide expression profiling platforms for which a variety of large-scale datasets are available in public repositories, including human B cell profiles on the U95Av2 platform [10], breast carcinoma and high-grade glioma profiles on the U133A and U133B platforms [12], and breast carcinoma, promyelocytic leukemia, prostate cancer, and glioblastoma profiles on the U133A platform [11,13,14], among many others. Cleaner improved cross-platform agreement between differential expression analyses of technical-replicate experiments from 41.6% to 61.2%, suggesting that nearly 50% of the gene expression measurements produced by these large-scale studies can be significantly improved by Cleaner analysis. The main ideas behind Cleaner are illustrated in Figure 1.

We compared Cleaner both to analyses using Affymetrix annotation and AffyProbeMiner annotation [9]. The latter is a recent effort focused on remapping and re-clustering microarray probes, and it compares favorably to other sequence-alignment centric efforts. We measured inter- and intra-microarray consistency by computing the correlation between repeated GeneAtlas gene profiling experiments using U133A, and by comparing gene sets identified as differentially down-regulated in centroblasts relative to naïve B cells using U95Av2 and U133plus2 platforms. We show that annotation by Cleaner significantly and systematically improved consistency across experiments and platforms, thus both improving accuracy of downstream analysis and simplifying the integration of experiments from multiple sources. To quantify these improvements, we performed quantitative reverse transcription real time PCR (qRT-PCR) validation of the expression of *FOXM1 *and *MYB *in human B cells, two genes that are mapped to multiple Affymetrix probe sets. Our experiments suggest that several of these probe-sets are conflicting and uninformative in the specific cellular context. As such, they should be disregarded when analyzing samples from naïve and centroblast B cells. Unlike other annotations, by pooling all consistent matching probes on the array, Cleaner annotation produced a single probe cluster per gene and gave definitive expression estimates that we validated here. In general, we showed that thousands of genes were associated with conflicting or uninformative Affymetrix probe sets in this B cell dataset, and Cleaner identified and resolved >95% of these instances.

Cleaner's approach is virtually technology-independent and can be easily adapted to clustering probes from other microarray platforms as well as short reads from deep-sequencing based approaches. For instance, probe clustering using data from exon-arrays will improve the identification of the specific isoforms that are differentially expressed across the samples, thus removing those that are not informative and significantly simplifying downstream analysis. Similarly, clustering short overlapping transcript fragments according to read multiplicity in deep-sequencing datasets may allow for improved transcript and exon-boundary detection, help estimate the frequency of splicing events, and help deconvolve and assign origin for reads with homology to several sites in the reference genome [15]. Finally, the substantial accuracy improvement achieved using Cleaner suggests that its use offers a unique opportunity to reevaluate inferences made from past Affymetrix gene expression profiles, which comprise 80% of the data-sets currently deposited in the Gene Expression Omnibus (GEO) [16], and points to Cleaner's potential impact on future microarray and deep-sequencing gene expression profile experiments. Cleaner, implemented in R and Python, is available for download from Califano Lab [17].

## Results

We begin by describing the Cleaner algorithm, and then show that Cleaner probe clusters improve consistency across technical replicate experiments and across platforms by eliminating biased and flawed probes. We conclude with a targeted study showing that probes in Affymetrix probe sets with inconsistent behavior are regrouped into consistent and informative probe clusters by Cleaner.

### The Cleaner algorithm

Cleaner proceeds by (a) remapping individual probes to the most recent RefSeq transcripts, (b) discarding probes mapped to multiple genes or incorrect regions, (c) computing correlation between all probe pairs on the same gene, and finally (d) organizing probes in clusters that are optimally intra-correlated within the specific context. A detailed description of each of these steps is given below.

#### Probe mapping to RefSeq genes

We mapped probe sequences to the transcripts in the RefSeq database [18] dating 11 December 2008 using ZOOM [19] and allowing for at most one mismatch per probe (each probe sequence matched at least 24 transcript positions). We matched against the positive orientation of RefSeq transcripts only. Probes that matched multiple non-overlapping genes were discarded, and each location in each matching transcript was annotated.

#### Building clean probe clusters

Based on probe mapping to RefSeq transcripts and corresponding genes, we constructed transcript-focused probe clusters in three steps: 1, quality control for individual probes; 2, clustering of correlated probes; and 3, testing of probe-cluster consistency. Probe clusters were used to create CDF files for assigning quantitative probe-cluster intensity by MAS5.

##### Step 1: probe consistency

We first established the consistency of each individual probe based on its correlation to other probes that were mapped to the same gene (neighbors) across microarray experiments. In this gene-focused approach, the readout obtained from any given probe is informative only if it is significantly correlated with other probes mapping to the same gene. First, probe readouts were quantile normalized to abstract away correlation among probes generated by inter-sample systematic bias. Then, the consistency score of each probe was set to the 90th percentile of computed Pearson correlation coefficients across its neighbors. Statistical significance was estimated on a gene-per-gene basis using a null distribution generated by computing the correlation between the probes mapping to the gene and 1,000 probes selected uniformly at random. Probes with consistency score corresponding to *P *> 0.01 were discarded (Figure 1a).

##### Step 2: probe clusters

Neighboring probes that map to isoforms that are differentiated by alternative splicing, RNA editing, or non-representative hybridization can produce readouts of different molecular species leading to poor quantitative intensity evaluation for the probe cluster. To account for RNA isoforms, we constructed transcript-focused probe clusters by performing a non-supervised, single-linkage hierarchical clustering of the probes using Pearson correlation coefficient as a distance measure. First, clusters were formed by iteratively breaking dendrogram edges that were significantly longer than the remainder of the edges in each level according to a one-tail *t*-test threshold of *P *< 10^-10 ^(Figure 1b, c). Then, we iteratively merged cluster pairs with distance significance greater than 0.001, where distance between clusters was defined as the distance between the closest elements across clusters, and significance was estimated using a null distribution of 1,000,000 distances between randomly selected probe pairs. For illustration, Figure 1d depicts the two probe clusters identified for MAX across three of its known isoforms.

##### Step 3: probe cluster consistency

Low information probe clusters, composed of few or dependent probes, were eliminated to reduce the false discovery rate (FDR). Overlapping probes account for 87.2% of the U95av2 remapped probes and 66.1% of the U133plus2 remapped probes, and they are affected by systematic bias resulting from common technical artifacts and cross-hybridization. These biases artificially improve pairwise correlations across expression profiles and can be estimated by conditioning on probe-overlap size. Figure 1e pictorially demonstrates that pairwise Pearson correlations between probes can be described as an exponential function of their overlap size; we found this significant behavior to hold true across platforms and experiments. To assign consistency scores for probe clusters, we derived a score for computing the contribution of each probe according to the size of its overlap with its upstream neighbor. Each probe contributed at most one point to the total score, and the contribution of a probe that overlaps another upstream probe was accounted for according to *s*(*x*):

where *x *is the shortest distance between probe starting positions across isoforms (position shift in Figure 1e); *a*, *b *and *c *are estimated by fitting *f*(*x*) to pairwise Pearson correlations for 1 ≤ *x *≤ 24. We estimated the probe-cluster FDR for each consistency score using permutation testing, where each probe cluster constructed after permuting sample labels (individually for each probe) was considered a false positive detection. For all experiments reported in this study, we set the minimum probe-cluster consistency score to *s*(*x*) ≥ 3. As shown in Figure 1f, this minimum score corresponds to FDR <5*e*-03 for the U95av2 B-cell samples.

#### Minimum sample size

To estimate the sample-size effect on Cleaner analysis, we randomly selected subsets from the 152 U95Av2 and 200 U133plus2 microarray experiments in B cells, and estimated the FDR when constructing probe clusters with Cleaner; we selected 20 samples per sample size. Results suggest that Cleaner is not effective for analyzing data derived from fewer than 20 microarray experiments. FDR and probe-cluster sizes showed no significant change for expression sets consisting of 40 or more microarray experiments, suggesting that full statistical power is obtained at this size (Figure S1 in Additional file 1). We note that we have obtained encouraging results using datasets with as few as 30 samples; these are described later in this section.

#### Probe sets generated by Cleaner for U95Av2 and U133plus2 platforms

Cleaner rejects probes due to poor matches to RefSeq transcripts and poor fit to a consistent probe cluster. Table 1 describes the total number of probes available, the number of probes retained by Cleaner, and the number of Cleaner probe clusters when measuring B-cell expression using U95Av2 and U133plus2. To demonstrate that similar efficiency was observed in other tissues, we provide the analogous information for lung, ovary, glioblastoma multiforme, prostate and breast carcinoma samples (Additional file 2). Poor gene matching, due to no- or multiple-gene homology, resulted in a loss of 23% and 47% of the probes for U95Av2 and U133plus2, respectively (Remap in Table 1). Probe and probe-cluster consistency analysis further discarded 48% and 67% of the RefSeq-mapped probes in U95Av2 and U133plus2, respectively. Cleaner eliminated most of the original probes, and represented approximately half of the probed genes by at least one probe cluster.

**Table 1:** Number of probes, probe sets, probe clusters and genes represented on two popular Affymetrix GeneChip microarrays

		Affymetrix	APM	Remap	Cleaner
U95Av2	Probes	200,073	163,939 (82%)	154,485 (77%)	83,862 (42%)
	Probe-sets	12,625	9,130 (72%)	8,410 (67%)	6,011 (48%)
	EntrezID	8,975	8,781 (98%)	8,410 (94%)	5,923 (66%)
					
U133plus2	Probes	594,532	326,265 (55%)	318,978 (54%)	153,960 (26%)
	Probe-sets	54,675	19,887 (36%)	18,303 (33%)	12,162 (22%)
	EntrezID	20,327	18,596 (91%)	18,303 (90%)	11,728 (58%)

We report the number of probes, probe-sets, probe clusters and unique EntrezIDs for the U95av2 and U133plus2 platforms according to Affymetrix, AffyProbeMiner (APM), Remap, and Cleaner annotation. Remap is the first stage of Cleaner, and here it is used to report the number of probes with homology to RefSeq transcripts and the number of probe sets and genes with at least one probe homolog. For simplicity of presentation, we refer to Remap and Cleaner probe clusters as probe sets in this table.

Of the probes discarded due to consistency issues, 55% and 58% were originally mapped to genes containing no consistent probes on U95Av2 and U133plus2 platforms, respectively (Figure S2 in Additional file 1), possibly reflecting the lack of detection of the given transcripts. In fact, we observed a relationship between expression intensity and probe-cluster consistency (Figure 1g). However, while low-intensity probe clusters are significantly more likely to be eliminated, low intensity on its own is not a sufficient requirement for rejection: many high-intensity probe clusters get discarded and low intensity probe clusters kept. In total, over 40% of the discarded probes were mapped to isoforms with consistent probe clusters, suggesting that individual probes mapped to expressed genes can be inconsistent due to technical bias (Figure S2 in Additional file 1). Finally, a small proportion of remapped probes aligned to RefSeq transcripts with a single mismatch, and Cleaner eliminated these imperfectly matching probes at a significantly higher rate than that of perfectly matching probes. Consequently, imperfectly matching probes accounted for a very small portion of the consistent probes in Cleaner probe clusters.

### Agreement across technical replicate experiments

**Figure 2:**
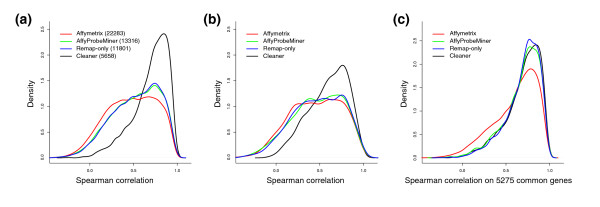
Consistency across technical replicate experiments. Density distributions for the Spearman correlation coefficient across technical replicates considering: (a) all the probe clusters generated by each method (size in parenthesis); (b) only the 1,000 probe clusters with the highest sample variation for each method; and (c) only probe clusters corresponding to genes that were detected by all methods. Cleaner probe clusters show dramatically better agreement across technical replicates even after pruning out low variability probe sets (b), and when restricting the comparison to genes that are present in all annotation strategies (c). Remap-only probe sets were statistically indistinguishable from AffyProbeMiner probe sets; both probe sets showed significantly better agreement across technical replicates than Affymetrix probe sets in (a, c), but the three were statistically indistinguishable after pruning out low sample variation probe sets (b).

Repeatability of experimental results is one of the basic requirements of any technology used for research and product development. We show that experimental repeatability is highly influenced by probe-set annotation quality. We measured Spearman correlation between replicate experiments from GeneAtlasV2 for 30 human tissue samples using Affymetrix, AffyProbeMiner and Cleaner annotation. Correlation coefficient distributions are given in Figure 2 and demonstrate that experimental replicate agreement shows a marked improvement when using Cleaner probe-cluster annotation. To eliminate potential circularity, Cleaner probe clusters were constructed in each of the 30 sample sets separately. In total, 79% of all consistent probes according to at least one of the annotations were included in probe clusters in both annotations. Correlations were computed for genes with exactly one probe cluster annotation.

To better quantify the distinct role of probe-remapping and probe-correlation analysis (consistency-testing), we included results taken at an intermediary step of the Cleaner algorithm (Remap-only in Figure 2), where RefSeq-mapped probes were used for probe-cluster construction. Results show that, without consistency-testing, the difference between Cleaner and AffyProbeMiner analyses is not statistically significant (at *P *≤ 0.05; Figure 2a). This suggests that Cleaner's improvements are mostly due to the elimination of inconsistent probes and to the construction of intrinsically consistent probe clusters rather than to an improved genome-probe mapping. In addition, to demonstrate that probe-set level pruning does not bridge the performance gap between Cleaner and the other annotations, we selected the 1,000 probe sets with highest coefficient of variation across samples for each annotation and repeated the comparison (Figure 2b); coefficient of variation-based pruning is commonly used to remove poorly informative probe sets from microarray expression experiments [20].

Finally, we restricted the comparison to genes with at least four annotated probes according to each annotation method; these genes had a sufficient number of consistent probes to generate Cleaner probe clusters and therefore they were expected to have accurate measurements according to all methods. Surprisingly, correlation coefficient distributions using Cleaner, AffyProbeMiner and Affymetrix annotation were significantly different. Remapping probes to RefSeq transcripts improved correlation across replicate experiments, and removing remapped inconsistent probes further increased correlation (Figure 2c). Our results suggest that Cleaner probe clusters are significantly more consistent across technical replicate experiments, and that the benefit of its probe-level selection and pruning cannot be achieved using probe-set level pruning.

### Consistency across platforms

**Figure 3:**
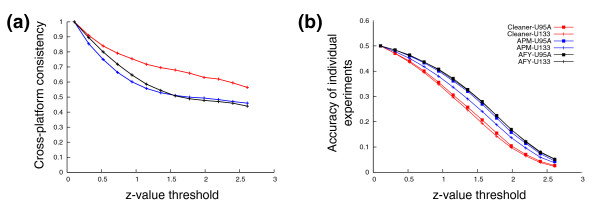
Cross-platform consistency for differential expression analysis. Comparison of cross-platform consistency (U95Av2 versus U133P2), and estimated individual accuracy of differential expression calls using Cleaner (red), AffyProbeMiner (blue) and Affymetrix (black) annotation. Comparisons are made as a function of the z-value threshold used for identifying differentially down-regulated genes (x-axis). (a) Cross-platform consistency was measured as the proportion of genes that are called down-regulated by both platforms to genes that are probed by both platforms and are called down-regulated by at least one of the platforms. (b) Accuracy of individual experiments was measured using FDR estimates from permutation testing, where all probe clusters scoring above threshold in the original data are called true positives and all probe clusters identified in permutated data are called false positives.

Differential expression analysis is routinely used to quantify cross-platform consistency [8,20]. To measure consistency, we identified differentially down-regulated genes in centroblast B-cell gene-expression profiles relative to naïve B-cell gene-expression profiles. Such genes may contribute to mature B-cell germinal-center formation. In order to discover them, samples in five biological replicates for naïve and for centroblast B cells were hybridized to Affymetrix GeneChip U95Av2 and later to U133plus2 microarrays [10,21]. We measured the consistency of differentially expressed gene sets across the two platforms, and the FDR of down-regulation calls in each platform under Affymetrix, AffyProbeMiner, and Cleaner probe-cluster annotation. Figure 3 shows that U133plus2-based analysis was consistently more accurate than U95Av2-based analysis, and that Cleaner produces significantly and dramatically more accurate intra- and inter-platform results.

To identify centroblast down-regulated gene candidates, we used a z-value cutoff of 2.33 (*P *< 0.01) for calling down-regulation together with an added 1.5-fold change requirement. Permutation testing estimates for the FDR in the U133plus2 analysis were 10.7%, 5.4% and 3.2% for Affymetrix, AffyProbeMiner, and Cleaner probe-cluster annotation, respectively. Focusing on genes probed by both U95Av2 and U133plus2 platforms, we identified 859 and 1,234 down-regulated genes in centroblasts by using Affymetrix annotations; 742 and 989 down-regulated genes by using AffyProbeMiner; and 677 and 801 down-regulated genes when using Cleaner. For Affymetrix annotation, 1,478 genes were called down-regulated by at least one of the platforms and 615 (41.6%) genes were called down-regulated in both platforms; this ratio improved to 550 of 1,181 (46.6%) for AffyProbeMiner, and to 562 of 919 (61.2%) for Cleaner. Finally, only 394 genes were called down-regulated by all annotations on all platforms. Note that enrichment results are independent of the actual number of differentially expressed genes identified in each method and each platform. U133plus2 included more probes, more probe sets, and more probe clusters and it was consistently more accurate. The most accurate results were produced using Cleaner, which defined the fewest probe clusters. To conclude, we note that according to differential expression analysis, expression consistency across platforms when using Cleaner annotation (61.2%) was 50% better than when using Affymetrix annotation (41.6%).

### Identification of biased probes

To demonstrate that Cleaner consistency testing is sufficient to identify biased and poorly designed probes, we focused on two types of probe features that are known to affect accuracy: G-spot probes and probes matching the transcript antisense. G spots have been shown to bias expression measurements [22], although not all probes containing G spots are flawed; we showed that Cleaner preferentially discards G-spot probes. Probes matching the antisense of transcripts are at best noisy and at worst hybridizing with the wrong gene; we temporarily included anti-sense probe alignments when remapping probes, and showed that Cleaner discards almost all antisense probes.

We used DME and motifclass [23] to identify patterns that are enriched in sequences of discarded probes relative to sequences of consistent probes. To ensure that discarded probes were truly individually inconsistent and were not discarded due to obsolete genomic annotation or poorly expressed target genes, we restricted the study to consistent probe-clusters corresponding to genes that had less than 20% probe rejection rates. The most enriched motifs identified were CGGGGG and GGG [G|A] [G|C]; both motifs were significantly (*P *< 0.001) enriched according to permutation testing, and CGGGGG had sites in 46% of the inconsistent probes and 20% of the consistent probes. This result suggests that patterns such as G spots are strongly correlated with probe bias, but may not be sufficient criteria for probe selection and pruning. Twenty percent of the consistent probes included a CGGGGG substring but still showed significant correlation to neighboring G-spot-free probes. Thus, correlation analysis discriminates between biased and faithful probes independent of the source of the bias and outperforms feature-specific analysis.

Both Affymetrix and AffyProbeMiner probe-set annotations include probes that match the antisense orientation of genes (see discussion about *FOXM1 *probe set 41324_g_at for an example). Cleaner does not permit antisense remapping; however, in order to test Cleaner's ability to identify inconsistent probes, we temporarily allowed antisense remapping. Antisense remapping of U95Av2 and U133plus2 probes identified 6,521 and 21,000 probes with unique homology to the reverse strand of RefSeq transcripts. Cleaner found that 6,013 (92%) of the U95Av2 and 18,511 (88%) of the U133plus2 antisense probes are inconsistent. For the majority of probes, antisense mapping should not produce clear and stable signals across profiles, and indeed Cleaner was able to recognize the vast majority of these poorly designed probes.

### Inconsistent behavior of Affymetrix probe sets

**Figure 4:**
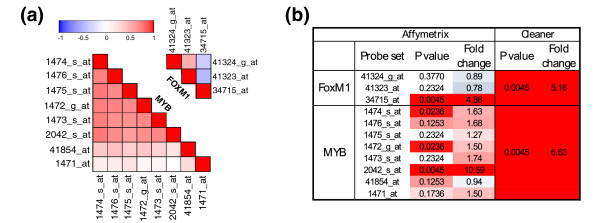
Inconsistent behavior of Affymetrix probe sets. (a) Correlation matrix for the Affymetrix annotated probe-sets associated with *FOXM1 *and *MYB *on the U95Av2 platform. The color scale, from blue to red, depicts the correlation (Pearson correlation coefficient) between probe sets. (b) Differential expression between centroblasts and naïve B cells as measured by *FOXM1 *and *MYB *probe-sets; *P*-values were estimated by U test and the color scale is used to emphasize significant differential expression and up-regulation.

U95Av2 and U133plus2 Affymetrix annotations include 2,168 and 10,895 genes with multiple probe-set definitions. Same-gene probe sets exhibit varying expression estimates: 3,840 of 4,344 and 35,873 of 43,285 probe-set pairs were positively correlated across B-cell samples as measured using U95Av2 and U133plus2 expression arrays. Of these, only 1,780 (46%) and 14,157 (39%) were significantly correlated (*P *< 0.01; Figure S3 in Additional file 1). Not surprisingly, Cleaner annotations include substantially fewer genes with multiple probe clusters: 89 and 541 for U95Av2 and U133plus2, respectively. Given reliable and consistent probe clustering, alternative probe sets can be used to capture variably expressed isoforms. However, when confidence in probe-set construction is low, the existence of poorly agreeing probe sets is not necessarily related to alternative isoforms. To demonstrate the challenge presented by multiple probe sets per gene, we focused on two genes associated with multiple probe sets in Affymetrix annotation. *FOXM1 *is represented by three probe sets in the U95Av2 platform, two of them showing a weak correlation across the B-cell samples and the third anti-correlated with the first two. MYB is associated with eight probe sets, six of them strongly correlated and two showing a weak correlation (Figure 4a). From these, only one probe set per gene was identified as up-regulated in centroblasts at *P *< 0.01 (Figure 4b). On the whole, only 207 (10%) and 1,452 (13%) of 2,168 and 10,895 genes profiled using U95Av2 and U133plus2 expression arrays had multiple differentially expressed probe sets, which is consistent with previously described poor agreements between probe sets that are associated with the same gene.

**Figure 5:**
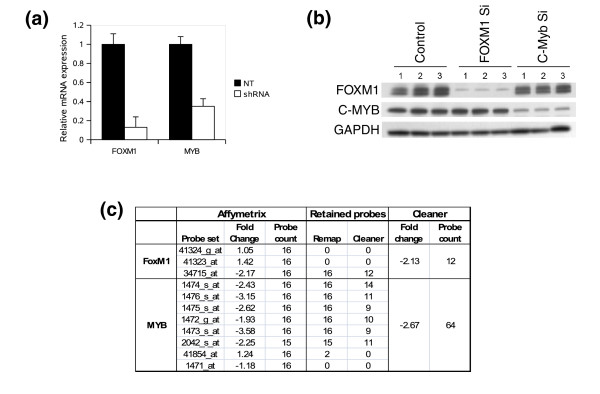
Probe-sets accurately reporting *FOXM1 *and *MYB *transcript levels. (a) mRNA levels for *FOXM1 *and *MYB *by qRT-PCR 24 h after lentiviral-mediated shRNA expression of a non-target sequence (NT; black bars); *FOXM1*- and *MYB*-specific sequences are shown by white bars. (b) FOXM1 and MYB protein levels by immunoblot 24 h after lentiviral-mediated shRNA expression of a non-target sequence (Control), and *FOXM1*- and *MYB*-specific sequences. (c) Affymetrix probe sets associated with *FOXM1 *and *MYB *transcripts fail to conclusively describe the effects of shRNA-mediated silencing. After *FOXM1 *and *MYB *silencing, some Affymetrix-annotated probe sets suggest mild increase in FOXM1 (two of three) and MYB expression (one of eight). Probe remapping to RefSeq transcripts and Cleaner annotation suggest that probes assigned to these probe sets either fail to match target genes or are inconsistent. As a result, Cleaner produced single probe clusters that correctly capture the shRNA-mediated knock-down validated by qRT-PCR.

To identify which of the Affymetrix probe sets and Cleaner probe clusters are accurately estimating *FOXM1 *and *MYB *gene expression, we knocked-down the two genes and compared their transcript levels using qRT-PCR and expression profiling on the U95Av2 platform. We used the Cleaner probe clusters constructed using the B cell sample data (Table 1). The knock-down of both genes was confirmed at the mRNA and protein levels by a qRT-PCR reaction designed to detect all known splicing variants and by immunoblot (Figure 5a, b). Results given in Figure 5c show that only one probe set for *FOXM1 *and five highly correlated probe sets for *MYB *(Figure 4a) agree with the qRT-PCR results at a two-fold threshold. Probe sets that indicated *FOXM1 *and *MYB *down-regulation in germinal center (Figure 4b) also indicated up-regulation after short hairpin RNA (shRNA)-mediated knock-down (Figure 5c). Cleaner probe clusters conclusively indicated up-regulation for the two genes in germinal center (Figure 4b), and down-regulation for the two genes after shRNA-mediated knock-down (Figure 5c).

Interestingly, the six *MYB *probe sets responding to *MYB *knock-down contributed to the Cleaner cluster, while all but two probes from the non-responding probe sets were discarded during the remapping process (Figure 5c). The two remapped probes for the conflicting *MYB *probe set 41854_at were discarded at a later stage by Cleaner consistency analysis. Similarly, the *FOXM1 *probe set responding to *FOXM1 *knock-down was the only one contributing to the Cleaner cluster, while all the probes for the remaining probe-sets were discarded during the remapping process (Figure 5c). An expanded probe-by-probe description of Figure 5c can be found in Additional file 3. Probe alignment locations to the two genes, as well as shRNA and qRT-PCR primer target locations, are given in Figure S4 in Additional file 1. Note that the *FOXM1 *probe set 41324_g_at aligns to the reverse complement (antisense) of the gene and is not included in the set of remapped probes in Figure 5c. We included the probe set in Figure S4 in Additional file 1 as an example for antisense remapping and, as expected, inclusion of these probes in the remapping stage only resulted in their elimination by Cleaner due to low consistency scores.

Finally, in Figure S5 in Additional file 1 we report on a breast-carcinoma-specific test comparing HER2 protein presence and *HER2 *mRNA expression estimates by three Affymetrix probe sets and one Cleaner probe cluster. HER2 protein was detected in 31 of the 129 samples [24], and using gene set enrichment analysis we showed that while estimates by all three Affymetrix probe sets are significantly correlated with the presence of the protein, the Cleaner probe cluster provides the closest estimates.

## Discussion

Large scale gene expression profiles are used for applications, including constructing high quality gene networks and interaction maps [10,11], improving the efficiency of drug target identification [25], developing diagnostic methods for disease stratification [26], and improving the understanding of the factors contributing to physiologic and pathologic differences between cellular phenotypes [21]. Nonetheless, repeatability of gene-expression measurements is still a major issue, with consistency of differential-expressed gene calling in repeated assays or across platforms below 50%. Worse, our results show that, on average, expression array probes that are overlapping in all but one position on the same transcript achieve Pearson Correlation below 0.85 (Figure 5e). These issues, which are related to probe-degeneracy, post-transcriptional/sample-specific modifications, and experimental sample preparation are broad and will continue to affect even deep-sequencing-based gene-expression profiling methods. Additionally, there are intrinsic limits for the consistency of even technical replicates due to experimental error. While existing methods operate well below that theoretical threshold, Cleaner achieves reproducibility that is closer to the theoretical limit and addresses most of the other issues by discarding non-informative probes without sacrificing dynamic range (that is, discarding low-intensity probes). Indeed, consistent low-intensity probes can be more informative than many inconsistent high-intensity ones. We suggest that transcript-level measurement-accuracy can be efficiently improved by clustering multiple informative probes, a design that will further benefit from the introduction of deep-sequencing-based approaches.

Technology that uses multiple probes per target to estimate expression can take advantage of large-scale gene expression profiling to improve accuracy. We showed that testing probe consistency across individual measurements helps identify biased or uninformative probes, leading to increased accuracy when estimating expression intensities on the transcript and gene levels. Using relatively simple statistics, we were able to resolve ambiguity and correct for bias with no concern for its source. Moreover, we showed that pruning measurement estimates for probe sets rather than for individual probes will inevitably lead to discarding high-quality probes because their aggregates include polluting poor-quality probes. In comparison, data analysis that is focused on individual probes can eliminate non-informative expression estimates for both probes and probe clusters, thus constructing clean clusters and simultaneously and efficiently reducing data dimensionality. This last point is crucial for improving the power of downstream data analysis such as biomarker discovery, phenotype classification, and reverse engineering of transcriptional networks [10,20,27,28].

Due to poor mapping to RefSeq genes, poor consistency, low gene expression or low variability across experiments, we discarded most of the individual probe measurements in each Affymetrix gene chip. We retained probe-cluster representations for approximately half of the genes that were originally included in the array design. The loss of probe-level data was offset by dramatic improvements in measurement accuracy for the remaining probed genes. Our analysis suggests that the vast majority of data discarded is at best uninformative for downstream analysis, as in the case of unexpressed transcripts, and at worst reduces the accuracy of otherwise good probe clusters, as in the case of inconsistent individual probes. Namely, if a probe is affected by systematic bias, then transcript intensity estimates that disregard this probe and are solely based on faithful probe measurements will be more accurate.

In this study we analyzed large-scale expression profiles obtained using Affymetrix microarrays, which have been used to produce a variety of large-scale, publicly available datasets. However, our methodology extends to other technologies that use multiple probes per target, including exon arrays and deep sequencing. Ideas developed here for expression arrays, including overlapping probe analysis and isoform identification, are easily adapted to resolve probe dependence in data produced by other technologies.

**Figure 6:**
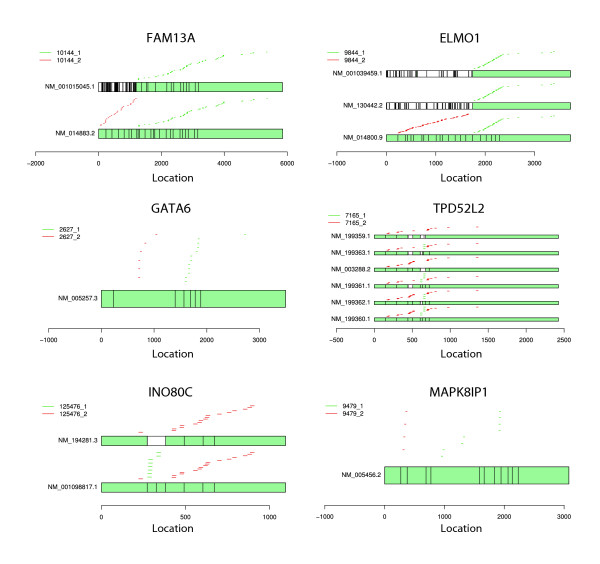
Cleaner probe clusters for six genes profiled on an exon array. Examples of putative Cleaner mRNA isoforms identified by using 55 human glial brain tumor samples hybridized on huex10stv1 Affymetrix exon arrays. The plots show the hybridization position for each probe on known and predicted mRNA isoforms for each gene.

Cleaner is directly applicable for analyzing Affymetrix exon arrays and is expected to produce alternative clusters for a larger set of genes. Figure 6 illustrates six examples of Cleaner probe clusters associated with known transcript isoforms (FAM13A, ELMO1, TPD52L2, INO80C) and Cleaner predicted isoforms (GATA6 and MAPK8IP1) using expression data from 55 human glial brain tumor samples hybridized on huex10stv1 Affymetrix exon arrays [29]. Note that alternative cluster probe positions for TPD52L2 and INO80C are interleaved. To emphasize that expression estimates for the alternative probe clusters are indeed very different, we compare expression estimates using the alternative clusters for the six genes in Figure S6 in Additional file 1.

Deep-sequencing reads are particularly amenable to the Cleaner approach. Understanding the relationship between reads and estimating the consistency of reads helps estimate target isoform concentration, and allows for eliminating or reevaluating biased and complex reads. Such reads include overlapping reads, reads that map to multiple transcripts, and reads that are biased due to features such as base composition or uneven copy number. In addition to technology-independent challenges addressed here, deep-sequencing data with sufficient coverage and dataset size will permit a more specific mixture resolution and the estimation of the contribution of individual sources producing observed read volumes. To extend the Cleaner method to deep-sequencing technology, partition RefSeq transcripts into equal-size bins, where the number of bins depends on the reads per transcript kilobase across samples. Each bin is scored based on the number of overlapping tags, and bins are treated as quasi-probes. Instead of using the distance between probes to score probe-cluster consistency, use the distance between bins. The rest of the algorithm directly follows Cleaner's current methodology.

## Conclusions

Genome-wide gene expression profile data suffer from technology-driven and technology-independent systematic bias. Measurement multiplicity, which is implicit to gene expression profiling technologies using multiple probes per target, can be used to assemble informative, transcript-specific probe-clusters that dramatically improve expression estimates in large cell-context-specific datasets. By harvesting the power of large-scale expression profiling we accounted for systematic biases regardless of their source. Our methods can be used to analyze both the large body of data in current repositories, and due to their technology-independent nature, they can be extended to construct transcript-specific probe-clusters using exon-array data and transcript-specific read-clusters in deep-sequencing data.

## Materials and methods

### Cell lines and cell culture conditions

We maintained ST486 and 293FT cells in Iscove's modified Dulbecco's medium (IMDM; Invitrogen, Carlsbad, CA, USA) and Dulbecco's minimum essential medium (DMEM; Invitrogen), respectively. Culture media was supplemented with 10% fetal bovine serum (Invitrogen) and 1% penicillin-streptomycin (Cellgro, Herndon, VA, USA).

### Lentiviral-mediated transduction

Control shRNA (SHC002), *FOXM1 *shRNA (TRCN0000015546) and *MYB *shRNA (TRCN0000040062) cloned into pLKO.1-puro lentiviral vector (Sigma, St Louis, MO, USA) were individually co-transfected with vesicular stomatitis virus glycoprotein envelope plasmid (280 ng) and Δ8.9 packaging vector (2.5 μg) into a subconfluent 100-mm plate of 293FT cells using Fugene 6 (Roche, Indianapolis, IN, USA). The viral particles were collected at 48 h and 72 h post-transfection and concentrated by ultracentrifugation in a Beckman SW28 rotor at 25,000 rpm for 1.5 h. ST486 cells (5 × 10^6^) were transduced with the viral particles in the presence of 8 μg/ml polybrene (Chemicon, Billerica, MA, USA) by centrifugation at 450 g for 1.5 h.

### Sample processing for qRT-PCR and microarrays

Total RNA was extracted with Trizol (Invitrogen) and purified by RNeasy kit (Qiagen, Valencia, CA, USA). For qRT-PCR, total RNA was reverse transcribed by QuantiTect Reverse Transcription kit (Qiagen) and SYBR-Green based qRT-PCR analysis was performed on an ABI7300 Real-time PCR system (Applied Biosystems, Foster City, CA, USA) using QuantiTect SYBR-Green kit (Qiagen). Relative quantification was performed with the 2^-ΔΔCt ^method [30], and was normalized by GAPDH expression by using the forward and reverse primers CACTGGGCCCTGACAACATC and TCACTCAGAGCTTGGGGTG for *FOXM1*, TGGGAGATGTGTGTTGTTGATG and TCCATGCAACAGTTCTGAGACC for *MYB*, and CACCCAGAAGACTGTGGATGGC and GTTCAGCTCAGGGATGACCTTGC for *GAPDH*. For microarray-based gene expression profiles, 5 μg of total RNA were processed following the manufacturer's instructions (Affymetrix, 701025 Rev.6), and 15 μg of fragmented and biotin-labeled cRNA were hybridized to HG-U95Av2 microarrays (Affymetrix, Santa Clara, CA, USA).

### Immunoblots

Whole cell lysates were prepared from ST486 cells by using RIPA buffer (Teknova, Hollister, CA, USA) with Complete Mini protease inhibitor cocktail (Roche). Proteins were fractionated by SDS-PAGE and analyzed by standard immunoblotting procedures using the following antibodies: anti-FOXM1 (sc-502), anti-MYB (sc-517) and anti-GAPDH (sc-32233), all from Santa Cruz (Santa Cruz, CA, USA).

### Expression profiles

Gene expression data include 102 B-cell samples profiled on U95A [10], 152 B-cell samples profiled on U95Av2 [10], 75 lung carcinoma samples on U95Av2 and U133plus2 [31,32], 51 and 295 ovarian cancer samples on U95Av2 and U133plus2 [33,34], 49 and 45 glioblastoma samples on U95Av2 (GSE13041) and U133plus2 [35,36], 88 and 154 prostate cancer samples on U95Av2 and U133plus2, 40 and 129 breast carcinoma samples on U95Av2 and U133plus2 [24,37], 200 B-cell samples profiled on U133plus2 [21], 60 samples from 30 human tissues profiled on U133A chips [38], and 55 human glial brain tumor samples hybridized on huex10stv1 Affymetrix exon arrays [29]. All samples were obtained from the GEO database [16] and GeneAtlasV2 [38]. A Bioconductor-based [39] implementation of MAS5 [40] was used to quantitatively estimate and normalize the intensity levels of probe clusters. Affymetrix annotation data were obtained from Bioconductor metadata packages hgu95av2.db and hgu133plus2.db v2.2.5 [39].

### Differential expression

We used a non-parametric U-test to identify down-regulated genes in centroblast B cells relative to naïve B cells. Comparisons were made using expression profiles from five biological replicates in each cell type. To identify a representative set of down-regulated genes per platform and annotation method, we used a z-value cutoff of 2.33 (*P *< 0.01) for calling differential expression together with a 1.5-fold change requirement. The fold change requirement was used in order to correct for the high expected FDR of this non-parametric test across thousands of probe clusters when using only five biological replicates for each cell type. The 1.5-fold decrease from naïve to centroblast B cells was based on the average intensities of the probe clusters after MAS5 normalization and *log*_2 _transformation. Analysis accuracy was measured using permutation testing repeated 20 times per annotation and platform, where the experimental source labels were shuffled for each probe cluster.

## Abbreviations

FDR: false discovery rate; GEO: Gene Expression Omnibus; qRT-PCR: quantitative reverse transcription real time PCR; shRNA: short hairpin RNA.

## Authors' contributions

MJA and PS developed the methodology, designed the research and wrote the manuscript; PR performed experiments and data analysis; AC designed the research and wrote the manuscript.

## Additional files

The following additional data are available with the online version of this paper: a PDF file containing Figures S1 to S6 (Additional file 1); Table S1 showing the number of probes, probe clusters, and genes represented on two popular Affymetrix Genechip microarrays after running Cleaner on different expression sets (Additional file 2); Table S2 showing the list of probes mapping to *FOXM1 *and *MYB *according to Affymetrix and Cleaner annotations (Additional file 3).

## Supplementary Material

Additional file 1 Figures S1 to S6. Click here for file

Additional file 2 Number of probes, probe clusters, and genes represented on two popular Affymetrix Genechip microarrays after running Cleaner on different expression sets. Click here for file

Additional file 3 Probes mapping to *FOXM1 *and *MYB *according to Affymetrix and Cleaner annotations. Click here for file

## References

[B1] Mecham B Klus G Strovel J Augustus M Byrne D Bozso P Wetmore D Mariani T Kohane I Szallasi Z Sequence-matched probes produce increased cross-platform consistency and more reproducible biological results in microarray-based gene expression measurements. Nucleic Acids Res 2004 32 E74 10.1093/nar/gnh071 15161944 PMC419626

[B2] Lossos I Czerwinski D Alizadeh A Wechser M Tibshirani R Botstein D Levy R Prediction of survival in diffuse large-B-cell lymphoma based on the expression of six genes. N Engl J Med 2004 350 1828 1837 10.1056/NEJMoa032520 15115829

[B3] Tan PK Downey TJ Spitznagel EL Xu P Fu D Dimitrov DS Lempicki RA Raaka BM Cam MC Evaluation of gene expression measurements from commercial microarray platforms. Nucleic Acids Res 2003 31 5676 5684 10.1093/nar/gkg763 14500831 PMC206463

[B4] Eisen MB Spellman PT Brown PO Botstein D Cluster analysis and display of genome-wide expression patterns. Proc Natl Acad Sci USA 1998 95 14863 14868 10.1073/pnas.95.25.14863 9843981 PMC24541

[B5] Hubbell E Liu W Mei R Robust estimators for expression analysis. Bioinformatics 2002 18 1585 1592 10.1093/bioinformatics/18.12.1585 12490442

[B6] Subramanian A Tamayo P Mootha VK Mukherjee S Ebert BL Gillette MA Paulovich A Pomeroy SL Golub TR Lander ES Mesirov JP Gene set enrichment analysis: a knowledge-based approach for interpreting genome-wide expression profiles. Proc Natl Acad Sci USA 2005 102 15545 15550 10.1073/pnas.0506580102 16199517 PMC1239896

[B7] Page G Coulibaly I Bioinformatic tools for inferring functional information from plant microarray data: tools for the first steps. Int J Plant Genomics 2008 2008 147563 18528524 10.1155/2008/147563 PMC2408683

[B8] Dai M Wang P Boyd AD Kostov G Athey B Jones EG Bunney WE Myers RM Speed TP Akil H Watson SJ Meng F Evolving gene/transcript definitions significantly alter the interpretation of GeneChip data. Nucleic Acids Res 2005 33 e175 10.1093/nar/gni179 16284200 PMC1283542

[B9] Liu H Zeeberg BR Qu G Koru AG Ferrucci A Kahn A Ryan MC Nuhanovic A Munson PJ Reinhold WC Kane DW Weinstein JN AffyProbeMiner: a web resource for computing or retrieving accurately redefined Affymetrix probe sets. Bioinformatics 2007 23 2385 2390 10.1093/bioinformatics/btm360 17660211

[B10] Basso K Margolin AA Stolovitzky G Klein U Dalla-Favera R Califano A Reverse engineering of regulatory networks in human B cells. Nat Genet 2005 37 382 390 10.1038/ng1532 15778709

[B11] Lamb J Crawford ED Peck D Modell JW Blat IC Wrobel MJ Lerner J Brunet JP Subramanian A Ross KN Reich M Hieronymus H Wei G Armstrong SA Haggarty SJ Clemons PA Wei R Carr SA Lander ES Golub TR The Connectivity Map: using gene-expression signatures to connect small molecules, genes, and disease. Science 2006 313 1929 1935 10.1126/science.1132939 17008526

[B12] Phillips HS Kharbanda S Chen R Forrest WF Soriano RH Wu TD Misra A Nigro JM Colman H Soroceanu L Williams PM Modrusan Z Feuerstein BG Aldape K Molecular subclasses of high-grade glioma predict prognosis, delineate a pattern of disease progression, and resemble stages in neurogenesis. Cancer Cell 2006 9 157 173 10.1016/j.ccr.2006.02.019 16530701

[B13] Wang Y Klijn JGM Zhang Y Sieuwerts AM Look MP Yang F Talantov D Timmermans M Meijer-van Gelder ME Yu J Jatkoe T Berns EMJJ Atkins D Foekens JA Gene-expression profiles to predict distant metastasis of lymph-node-negative primary breast cancer. Lancet 2005 365 671 679 15721472 10.1016/S0140-6736(05)17947-1

[B14] Cancer Genome Atlas Research Network Comprehensive genomic characterization defines human glioblastoma genes and core pathways. Nature 2008 455 1061 1068 10.1038/nature07385 18772890 PMC2671642

[B15] Mortazavi A Williams BA McCue K Schaeffer L Wold B Mapping and quantifying mammalian transcriptomes by RNA-Seq. Nat Methods 2008 5 621 628 10.1038/nmeth.1226 18516045 PMC13303166

[B16] Edgar R Domrachev M Lash AE Gene Expression Omnibus: NCBI gene expression and hybridization array data repository. Nucl Acids Res 2002 30 207 210 10.1093/nar/30.1.207 11752295 PMC99122

[B17] The Cleaner R-package http://wiki.c2b2.columbia.edu/califanolab/index.php/Cleaner

[B18] Pruitt KD Maglott DR RefSeq and LocusLink: NCBI gene-centered resources. Nucl Acids Res 2001 29 137 140 10.1093/nar/29.1.137 11125071 PMC29787

[B19] Lin H Zhang Z Zhang MQ Ma B Li M ZOOM! Zillions of oligos mapped. Bioinformatics 2008 24 2431 2437 10.1093/bioinformatics/btn416 18684737 PMC2732274

[B20] MAQC Consortium Shi L Reid LH Jones WD Shippy R Warrington JA Baker SC Collins PJ de Longueville F Kawasaki ES Lee KY Luo Y Sun YA Willey JC Setterquist RA Fischer GM Tong W Dragan YP Dix DJ Frueh FW Godsaid FM Herman D Jensen RV Johnson CD Lovenhofer EK Puri RK Schrf U Thierry-Mieg J Wang C The MicroArray Quality Control (MAQC) project shows inter- and intraplatform reproducibility of gene expression measurements. Nat Biotechnol 2006 24 1151 1161 10.1038/nbt1239 16964229 PMC3272078

[B21] Basso K Saito M Sumazin P Margolin AA Wang K Lim W-K Alvarez MJ Kitagawa Y Schneider C Califano A Dalla-Favera R Integrated biochemical and computational approach identifies BCL6 direct target genes controlling multiple pathways in normal germinal-center B cells. Blood 2009 in press 19965633 10.1182/blood-2009-06-227017 PMC2817639

[B22] Upton GJ Langdon WB Harrison AP G-spots cause incorrect expression measurement in Affymetrix microarrays. BMC Genomics 2008 9 613 10.1186/1471-2164-9-613 19094220 PMC2628396

[B23] Smith AD Sumazin P Zhang MQ Tissue-specific regulatory elements in mammalian promoters. Mol Syst Biol 2007 3 73 17224917 10.1038/msb4100114 PMC1800356

[B24] Lu X Wang Z Iglehart J Zhang X Richardson A Predicting features of breast cancer with gene expression patterns. Breast Cancer Res Treat 2008 108 191 201 10.1007/s10549-007-9596-6 18297396

[B25] Butte A Tamayo P Slonim D Golub T Kohane I Discovering functional relationships between RNA expression and chemotherapeutic susceptibility using relevance networks. PNAS 2000 97 12182 12186 10.1073/pnas.220392197 11027309 PMC17315

[B26] Hoshida Y Villanueva A Kobayashi M Peix J Chiang DY Camargo A Gupta S Moore J Wrobel MJ Lerner J Reich M Chan JA Glickman JN Ikeda K Hashimoto M Watanabe G Daidone MG Roayaie S Schwartz M Thung S Salvesen HB Gabriel S Mazzaferro V Bruix J Friedman SL Kumada H Llovet JM Golub TR Gene expression in fixed tissues and outcome in hepatocellular carcinoma. N Engl J Med 2008 359 1995 2004 10.1056/NEJMoa0804525 18923165 PMC2963075

[B27] Margolin A Nemenman I Basso K Wiggins C Stolovitzky G Favera R Califano A ARACNE: An Algorithm for the Reconstruction of Gene Regulatory Networks in a Mammalian Cellular Context. BMC Bioinformatics 2006 7 S7 10.1186/1471-2105-7-S1-S7 16723010 PMC1810318

[B28] van't Veer LJ Bernards R Enabling personalized cancer medicine through analysis of gene-expression patterns. Nature 2008 452 564 570 10.1038/nature06915 18385730

[B29] French P Peeters J Horsman S Duijm E Siccama I Bent M van den Luider T Kros J Spek P van der Sillevis Smitt P Identification of differentially regulated splice variants and novel exons in glial brain tumors using exon expression arrays. Cancer Res 2007 67 5635 5642 10.1158/0008-5472.CAN-06-2869 17575129

[B30] Livak KJ Schmittgen TD Analysis of relative gene expression data using real-time quantitative PCR and the 2(-Delta Delta C(T)) Method. Methods 2001 25 402 408 10.1006/meth.2001.1262 11846609

[B31] Stearman R Dwyer-Nield L Zerbe L Blaine S Chan Z Bunn PJ Johnson G Hirsch F Merrick D Franklin W Baron A Keith R Nemenoff R Malkinson A Geraci M Analysis of orthologous gene expression between human pulmonary adenocarcinoma and a carcinogen-induced murine model. Am J Pathol 2005 167 1763 1775 16314486 10.1016/S0002-9440(10)61257-6 PMC1613183

[B32] Ding L Getz G Wheeler D Mardis E McLellan M Cibulskis K Sougnez C Greulich H Muzny D Morgan M Fulton L Fulton R Zhang Q Wendl M Lawrence M Larson D Chen K Dooling D Sabo A Hawes A Shen H Jhangiani S Lewis L Hall O Zhu Y Mathew T Ren Y Yao J Scherer S Clerc K Somatic mutations affect key pathways in lung adenocarcinoma. Nature 2008 455 1069 1075 10.1038/nature07423 18948947 PMC2694412

[B33] Rusy K Temporal lobectomy: a promising alternative. J Neurosci Nurs 1991 23 320 324 1835997 10.1097/01376517-199110000-00009

[B34] Tothill R Tinker A George J Brown R Fox S Lade S Johnson D Trivett M Etemadmoghadam D Locandro B Traficante N Fereday S Hung J Chiew Y Haviv I Gertig D DeFazio A Bowtell D Novel molecular subtypes of serous and endometrioid ovarian cancer linked to clinical outcome. Clin Cancer Res 2008 14 5198 5208 10.1158/1078-0432.CCR-08-0196 18698038

[B35] Sun L Hui A Su Q Vortmeyer A Kotliarov Y Pastorino S Passaniti A Menon J Walling J Bailey R Rosenblum M Mikkelsen T Fine H Neuronal and glioma-derived stem cell factor induces angiogenesis within the brain. Cancer Cell 2006 9 287 300 10.1016/j.ccr.2006.03.003 16616334

[B36] Lee Y Scheck A Cloughesy T Lai A Dong J Farooqi H Liau L Horvath S Mischel P Nelson S Gene expression analysis of glioblastomas identifies the major molecular basis for the prognostic benefit of younger age. BMC Med Genomics 2008 1 52 10.1186/1755-8794-1-52 18940004 PMC2596165

[B37] Umemura S Shirane M Takekoshi S Kusakabe T Itoh J Egashira N Tokuda Y Mori K Osamura Y Overexpression of E2F-5 correlates with a pathological basal phenotype and a worse clinical outcome. Br J Cancer 2009 100 764 771 10.1038/sj.bjc.6604900 19259095 PMC2653774

[B38] Su AI Wiltshire T Batalov S Lapp H Ching KA Block D Zhang J Soden R Hayakawa M Kreiman G Cooke MP Walker JR Hogenesch JB A gene atlas of the mouse and human protein-encoding transcriptomes. Proceedings of the National Academy of Sciences of the United States of America 2004 101 6062 6067 10.1073/pnas.0400782101 15075390 PMC395923

[B39] Gentleman RC Carey VJ Bates DM Bolstad B Dettling M Dudoit S Ellis B Gautier L Ge Y Gentry J Hornik K Hothorn T Huber W Iacus S Irizarry R Leisch F Li C Maechler M Rossini AJ Sawitzki G Smith C Smyth G Tierney L Yang JY Zhang J Bioconductor: open software development for computational biology and bioinformatics. Genome Biology 2004 5 R80 10.1186/gb-2004-5-10-r80 15461798 PMC545600

[B40] Irizarry RA Bolstad BM Collin F Cope LM Hobbs B Speed TP Summaries of Affymetrix GeneChip probe level data. Nucleic Acids Research 2003 31 e15 10.1093/nar/gng015 12582260 PMC150247

