# Supplementary material for: Correlating measurements across samples improves accuracy of large-scale expression profile experiments
Source: Genome Biol. 2009 Dec 30;10(12):R143. doi: 10.1186/gb-2009-10-12-r143 (PMC2812950; doi:10.1186/gb-2009-10-12-r143)
Supplement: Additional file 1 — Figures S1 to S6. [file gb-2009-10-12-r143-S1.pdf]

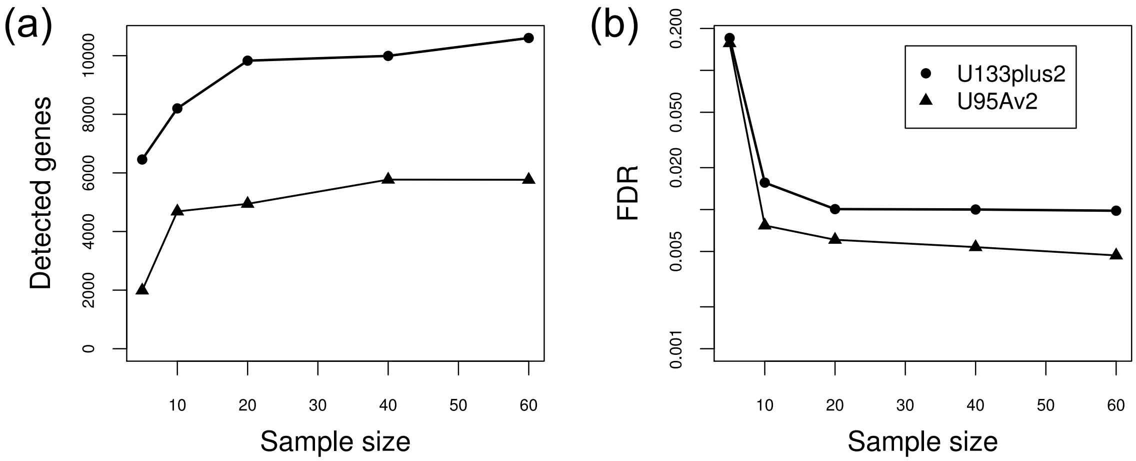

Supplementary Figure 1 Effect of sample size on Cleaner consistency.
(**a**) Number of detected probe clusters for variable-size randomly-selected subsets of the 152 and 200 microarray expression experiments on the U95av2 and U133plus2 platforms. (**b**) False discovery rate associated with subsets from panel A were estimated by permutation testing.

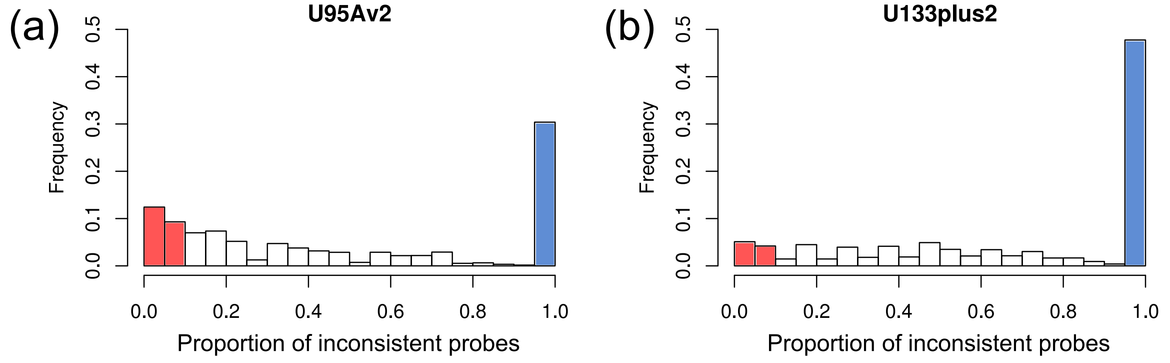

Supplementary Figure 2 Inconsistent probe distribution across probe sets. Frequency of Cleaner probe clusters with variable proportion of inconsistent probes for 50 B-cell samples hybridized to (**a**) U95Av2 and (**b**) U133plus2 gene chips. The majority of inconsistent (discarded) probes were mapped to genes with no consistent probes (blue), but 20% and 10% of inconsistent probes in U95Av2 and U133plus2 (red) were mapped to probe clusters with over 90% probe consistency rates.

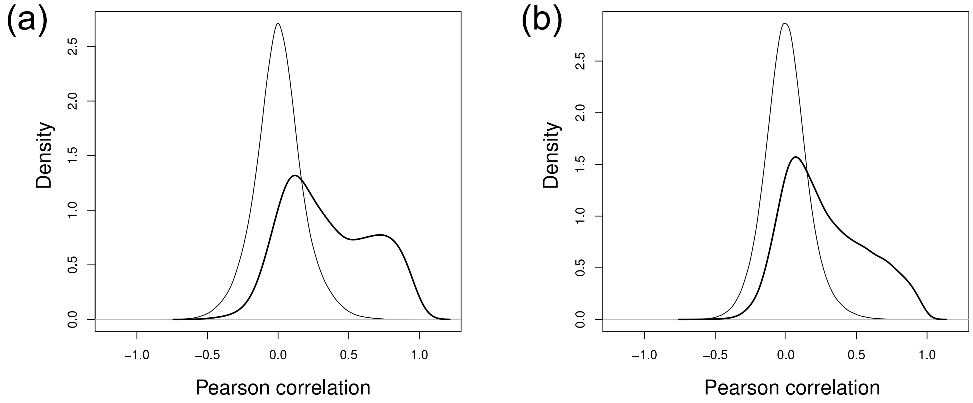

Supplementary Figure 3 Inconsistent behavior of Affymetrix probe-sets.
Correlation between Affymetrix probe-sets that map to the same gene for (**a**) 254 B-cell samples profiled on U95Av2 chips; and (**b**) 201 B-cell samples profiled on U133plus2 chips. Shown is the density distribution for all pairwise pearson correlation coefficients between probe-sets annotated with the same gene entrezID (thick line), and the null distribution computed by shuffling the probe-set labels (thin line).

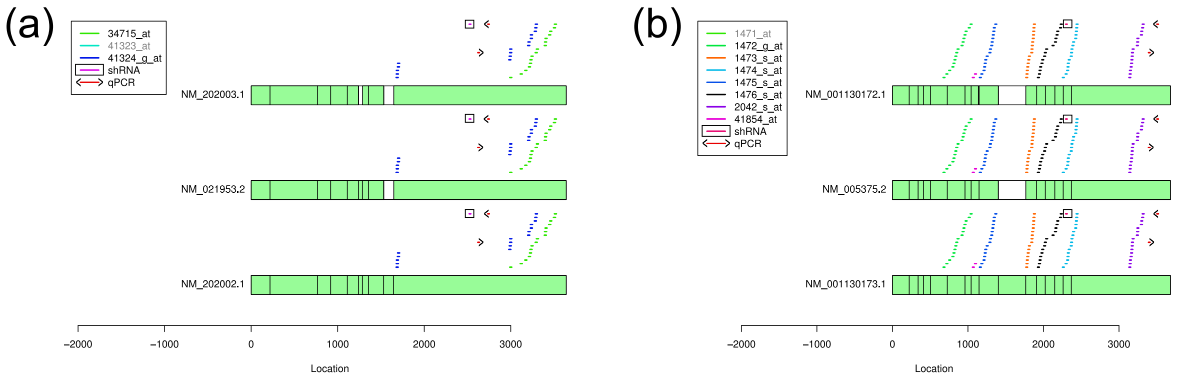

Supplementary Figure 4 Location of probes, shRNA, and qPCR primers in FOXM1 and MYB transcripts isoforms.
Schema of the known transcript isoforms for FOXM1 (**a**) and MYB (**b**) obtained from RefSeq database showing the mapping location for Affymetrix probe-sets. Similarly, target locations within the transcripts for shRNAs and qPCR primers are shown. Affymetrix `41324_g_at` probes mapping to the antisense of FoxM1, are rejected during remapping, and are shown here for illustration purposes only. Probes for `41323_at` and `1471_at` (grey letters) could not be mapped to RefSeq genes.

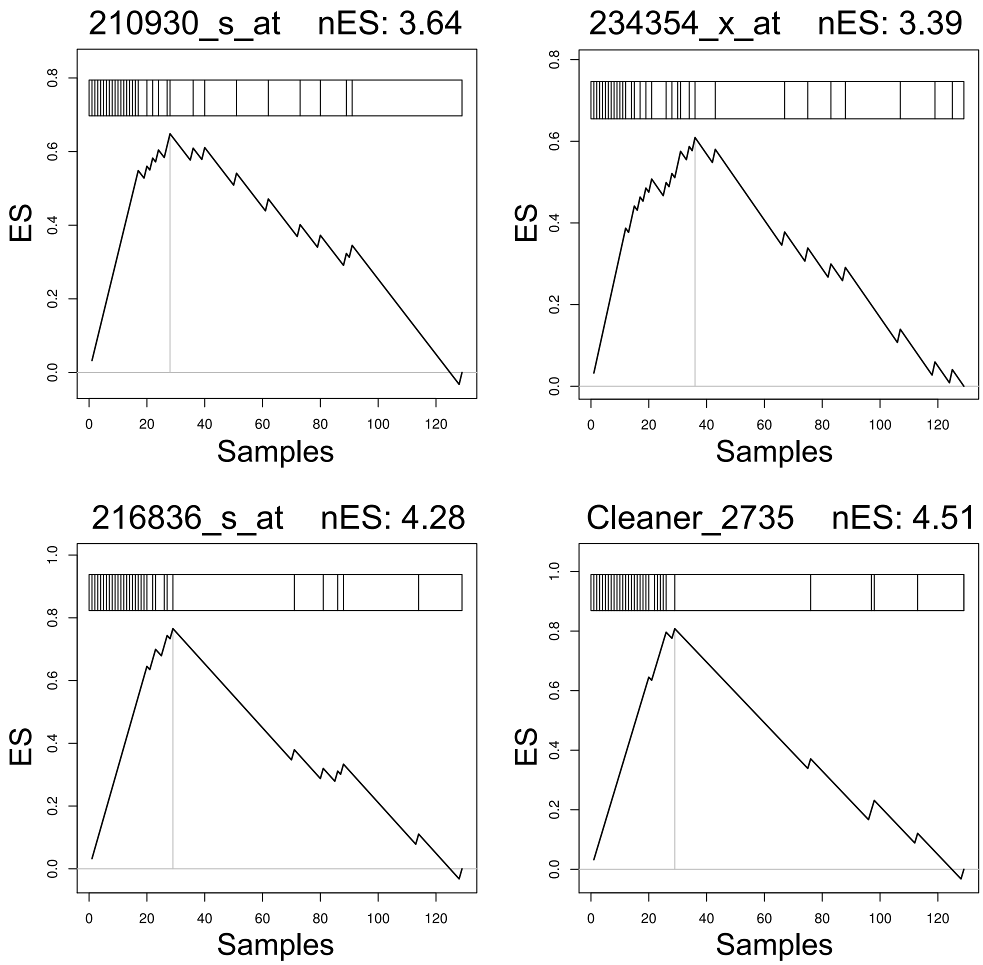

Supplementary Figure 5 Enrichment analysis of HER2-protein positive samples on the mRNA levels measured by 3 Affymetrix probe sets: 210930_s_at, 234354_x_at and 216836_s_at, and the unique Cleaner probe set 2735. 129 human breast carcinoma samples were rank-sorted according to HER2 probe-set signal levels and the enrichment of 31 HER2 protein positive samples (indicated by the barcode-like plot) was estimated by Gene Set Enrichment Analysis. Shown is the running-sum curve and the normalized enrichment score (nES) is indicated at the top of each panel. The cleaner probe cluster showed the highest enrichment score.

Alternatively, we computed the odds ratio of the HER2 positive samples among the top 31 samples. The Affymetrix probe-sets 210930_s_at, 234354_x_at and 216836_s_at showed odds ratios of 23.17, 17.82 and 59.52, respectively; while the odds ratio for the Cleaner probe cluster was 88.46.

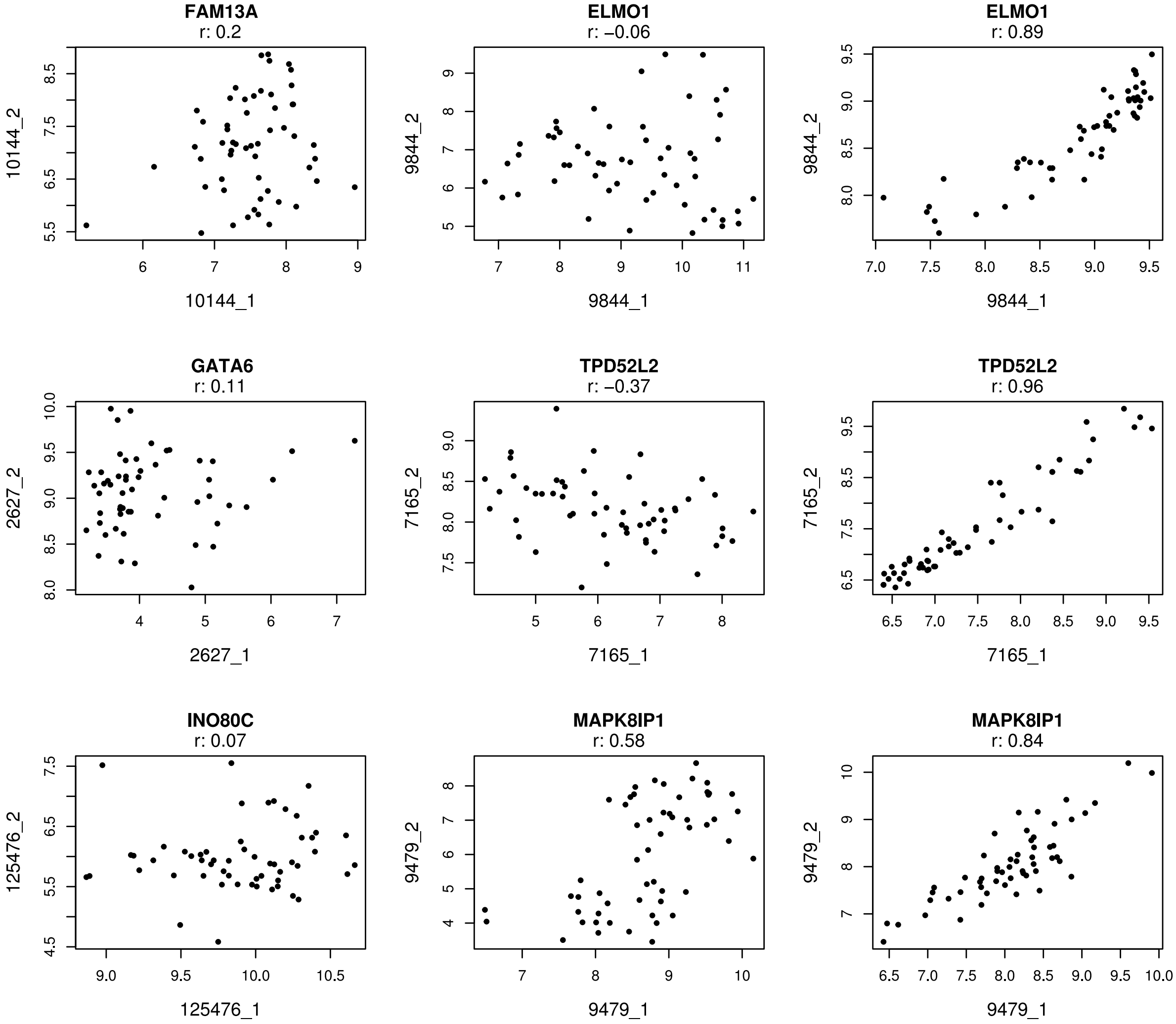

Supplementary Figure 6 Correlation between probe-clusters for 6 examples of putative mRNA isoforms identified by Cleaner from 55 human glial brain tumor samples hybridized on huex10stv1 Affymetrix exon arrays (GSE9385). The plots on the left two columns show the probe-cluster intensity after rma normalization for two probe-clusters mapping to the same gene (entrezID). Plots in the right column show the correlation between two probe-clusters generated by randomly selecting the probes from one of the probe clusters (9844_1, 7165_2 and 9479_1). Expression estimates according to these random partitions of Cleaner clusters are strongly correlated, while expression estimates according to altenative clusters show a considerably weaker correlation.
