# Supplementary material for: Correlating measurements across samples improves accuracy of large-scale expression profile experiments
Source: Genome Biol. 2009 Dec 30;10(12):R143. doi: 10.1186/gb-2009-10-12-r143 (PMC2812950; doi:10.1186/gb-2009-10-12-r143)
Supplement: Additional file 2 — Number of probes, probe clusters, and genes represented on two popular Affymetrix Genechip microarrays after running Cleaner on different expression sets. [file gb-2009-10-12-r143-S2.pdf]

Supplementary Table 1: Number of probes, probe clusters, and genes represented on two popular Affymetrix Genechip microarray after running Cleaner on different expression-sets. The proportion of probes, probe clusters and genes (EntrezID) compared to the current Affymetrix annotation is shown between parenthesis. Cleaner was ran on 152 and 200 B-cell samples hybridized on U95Av2 and U133plus2, respectively (Basso et.al. Nat. Genetics 2005, Basso et.al. Blood 2009); 75 lung carcinoma samples on U95Av2 (GSE2514 and GSE6253) and 75 on U133plus2 (GSE12667); 51 ovarian cancer samples on U95Av2 (GSE8057) and 295 on U133plus2 (GSE9899); 49 glioblastoma samples on U95Av2 (GSE13041) and 45 on U133plus2 (GSE4290); 88 prostate cancer samples on U95Av2 (GSE1431) and 154 on U133plus2 (GSE17951); 40 breast carcinoma samples on U95Av2 (GSE6367) and 129 on U133plus2 (GSE5460).

Platform		B cell		Lung		Ovary		GBM		Prostate		Breast	
U95Av2	Probes	83862	(42%)	51297	(26%)	41702	(21%)	43094	(22%)	41637	(21%)	61258	(31%)
	Probe clusters	6011	(48%)	4860	(38%)	4343	(34%)	4280	(34%)	4247	(34%)	5189	(41%)
	EntrezID	5923	(66%)	4780	(53%)	4193	(47%)	4229	(47%)	4224	(47%)	5118	(57%)
U133plus2	Probes	153960	(26%)	205597	(35%)	183864	(31%)	140031	(24%)	197675	(33%)	163868	(28%)
	Probe clusters	12162	(22%)	14894	(27%)	15376	(28%)	12656	(23%)	16753	(31%)	13205	(24%)
	EntrezID	11728	(58%)	14620	(72%)	14241	(70%)	11817	(58%)	14592	(72%)	12734	(63%)
