# Supplementary material for: Correlating measurements across samples improves accuracy of large-scale expression profile experiments
Source: Genome Biol. 2009 Dec 30;10(12):R143. doi: 10.1186/gb-2009-10-12-r143 (PMC2812950; doi:10.1186/gb-2009-10-12-r143)
Supplement: Additional file 3 — Probes mapping to FOXM1 and MYB according to Affymetrix and Cleaner annotations. [file gb-2009-10-12-r143-S3.pdf]

Supplementary Table 2 List of probes mapping to FOXM1 and MYB according to Affymetrix and Cleaner annotations.
Columns show the probe-set defined by Affymetrix, probe sequences, mapping to current RefSeq database (Remap), probe-clusters generated by Cleaner (Cleaner), and consistency score for each probe (consistency). Consistency scores lower than the 99 percentile of the permutation-based null distribution, 0.632 for FOXM1 and 0.532 for MYB, are shown between square brackets. (*) Probes removed by the clustering analysis. (^{RC}) Map to the reverse complement.

probe_set	Probe #	sequence	Remap	Cleaner	consistency
		FOXM1			
	1	CTTTGCAGGGTGGTCCGTGTAAATA	FOXM1	2305.1	0.84
	2	GGTTTCAATTGACTTCTGTTCCTTG	FOXM1	2305.1	0.91
	3	GCAGTGCACGGTTTCTTCCAGGCTG	FOXM1	2305.1	0.93
	4	GTTCTTCACTGCAGGGACCCAGACA	FOXM1	2305.1	0.9
	5	AGGGACCCAGACAAGTGGATCTGCT	FOXM1	-	0.65*
	6	CCAGACAAGTGGATCTGCTTGCCAG	FOXM1	2305.1	0.83
	7	ATCTGCTTGCCAGAGTCCTTTTTGC	FOXM1	2305.1	0.89
34715_at	8	TTCCAAGTCAGCTTTCCTGCAAGAA	FOXM1	2305.1	0.94
	9	TCCTGCAAGAAGAAATCCTGGTTAA	FOXM1	2305.1	0.76
	10	TGTGGGTGCCCAGATGTGCGCTATT	FOXM1	2305.1	0.94
	11	GGGTGCCCAGATGTGCGCTATTAGA	FOXM1	2305.1	0.94
	12	CCAGATGTGCGCTATTAGATGTTTC	FOXM1	2305.1	0.91
	13	TCATACCAGGGAGACTGGCATTGAC	FOXM1	2305.1	0.76
	14	GACTGGCATTGACGAGAACTCAGGT	FOXM1	2305.1	0.93
	15	AAAGGGCCCCTGACCTGCCTGGCTT	FOXM1	-	[0.37]
	16	GACCTGCCTGGCTTCCTTAGCTTGC	FOXM1	-	[0.55]
	17	CAAAGATCAGGGAAGGCTGGATTTC	-	-	-
	18	AAGGCTGGATTTCTTCCTCCTTGAT	FOXM1^{RC}	-	[0.51]
	19	CCTCAGCTAGCAGCACCTGAAAGGG	-	-	-
	20	AGCTAGCAGCACCTGAAAGGGAAAC	-	-	-
	21	AGCAGCACCTGAAAGGGAAACAGAG	-	-	-
	22	ATAAGGTGAACCAACGGTCACCAGA	-	-	-
	23	TAAGGTGAACCAACGGTCACCAGAC	-	-	-
41323_at	24	GTGAACCAACGGTCACCAGACAGGA	-	-	-
	25	CAACGGTCACCAGACAGGACGCACA	-	-	-
	26	TCACCAGACAGGACGCACAAAAATA	-	-	-
	27	CAGACAGGACGCACAAAAATATCAC	-	-	-
	28	AGGACGCACAAAAATATCACATACG	-	-	-
	29	GCACAAAAATATCACATACGGGTTC	-	-	-
	30	ATCACATACGGGTTCTGATCCTCTT	-	-	-
	31	GGGTTCTGATCCTCTTTGTGTCGTT	-	-	-

probe_set	Probe #	sequence	Remap	Cleaner	consistency
	32	CTGATCCTCTTTGTGTCGTTTTGAA	-	-	-
	33	TATTTACACGGACCACCCTGCAAAG	$FOXM1^{RC}$	-	[0.51]
	34	ATTTACACGGACCACCCTGCAAAGA	$FOXM1^{RC}$	-	[0.57]
	35	CACGGACCACCCTGCAAAGATCAGG	$FOXM1^{RC}$	-	[0.53]
	36	ACCACCCTGCAAAGATCAGGGAAGG	$FOXM1^{RC}$	-	[0.57]
	37	AACAGGAGTTTCTCCTCTTTCCCTG	$FOXM1^{RC}$	-	[0.46]
	38	CAGGAGTTTCTCCTCTTTCCCTGGT	$FOXM1^{RC}$	-	[0.45]
	39	CTCTTTCCCTGGTCCTGCAGAAGAA	$FOXM1^{RC}$	-	[0.52]
41324_g_at	40	TCTTTCCCTGGTCCTGCAGAAGAAA	$FOXM1^{RC}$	-	[0.39]
	41	TTTCCCTGGTCCTGCAGAAGAAAGA	$FOXM1^{RC}$	-	[0.37]
	42	TCTTCTTGCAGGAAAGCTGACTTGG	$FOXM1^{RC}$	-	[0.17]
	43	TGCAGGAAAGCTGACTTGGAAACAC	$FOXM1^{RC}$	-	[0.32]
	44	AAAGCTGACTTGGAAACACGGGGAG	$FOXM1^{RC}$	-	[0.28]
	45	GGACTCTGGCAAGCAGATCCACTTG	$FOXM1^{RC}$	-	[0.13]
	46	GACTCTGGCAAGCAGATCCACTTGT	$FOXM1^{RC}$	-	[0.44]
	47	GGGTCCCTGCAGTGAAGAACCCAAG	$FOXM1^{RC}$	-	[0.5]
	48	CCCTGCAGTGAAGAACCCAAGATCC	$FOXM1^{RC}$	-	[0.48]
			MYB		
	49	CCAACTGGGATGGCTCCTTGTGCTT	-	-	-
	50	ACTGGGATGGCTCCTTGTGCTTTGC	-	-	-
	51	GGGATGGCTCCTTGTGCTTTGCAAC	-	-	-
	52	ATGGCTCCTTGTGCTTTGCAACATA	-	-	-
	53	GCTCCTTGTGCTTTGCAACATACAT	-	-	-
	54	CCTTGTGCTTTGCAACATACATAGT	-	-	-
	55	AGCAGGTGCTACCATTATGTGGGCC	-	-	-
1471_at	56	AGGTGCTACCATTATGTGGGCCATT	-	-	-
	57	TGCTACCATTATGTGGGCCATTACT	-	-	-
	58	GGCCATTACTGAATTCTGACATCTT	-	-	-
	59	CATTACTGAATTCTGACATCTTTAG	-	-	-
	60	TACTGAATTCTGACATCTTTAGCGA	-	-	-
	61	TGAATTCTGACATCTTTAGCGACTG	-	-	-
	62	ATTCTGACATCTTTAGCGACTGGGC	-	-	-
	63	ACATCTTTAGCGACTGGGCAGCCAA	-	-	-
	64	ACTGGGCAGCCAACTGGGATGGCTC	-	-	-
	65	GCACACAAGAGACTGGGGAACAGAT	MYB	-	[0.36]
	66	AAAGCTACTGCCTGGACGAACTGAT	MYB	4602.1	0.79
	67	AACCACTGGAATTCTACAATGCGTC	MYB	4602.1	0.86
	68	GTTATCTGCAGGAGTCTTCAAAAGC	MYB	4602.1	0.7
	69	TCTTCAAAAGCCAGCCAGCCAGCAG	MYB	-	[0.19]
	70	GCCAGCCAGCAGTGGCCACAAGCTT	MYB	-	[0.25]
	71	TGGCCACAAGCTTCCAGAAGAACAG	MYB	4602.1	0.61

probe_set	Probe #	sequence	Remap	Cleaner	consistency
1472_g_at	72	TCCAGAAGAACAGTCATTTGATGGG	MYB	-	[0.21]
	73	TTGATGGGTTTTGCTCAGGCTCCGC	MYB	4602.1	0.64
	74	GCCCACTGTTAACAACGACTATTCC	MYB	4602.1	0.79
	75	ACGACTATTCCTATTACCACATTTC	MYB	4602.1	0.87
	76	TATTACCACATTTCTGAAGCACAAA	MYB	4602.1	0.72
	77	TGAAGCACAAAATGTCTCCAGTCAT	MYB	-	[0.47]
	78	CTCCAGTCATGTTCCATACCCTGTA	MYB	-	[0.39]
	79	TAGTCAATGTCCCTCAGCCAGCTGC	MYB	-	[0.53]
	80	CGCAGCCATTCAGAGACACTATAAT	MYB	4602.1	0.74
1473_s_at	81	TTCTTAAACACTTCCAGTAACCATG	MYB	4602.1	0.72
	82	TAAACACTTCCAGTAACCATGAAAA	MYB	4602.1	0.72
	83	CACTTCCAGTAACCATGAAAACTCA	MYB	4602.1	0.73
	84	CAGTAACCATGAAAACTCAGACTTG	MYB	-	0.61*
	85	CCATGAAAACTCAGACTTGGAAATG	MYB	-	[0.29]
	86	ACTCAGACTTGGAAATGCCTTCTTT	MYB	4602.1	0.8
	87	TTGGAAATGCCTTCTTTAACTTCCA	MYB	-	0.58*
	88	TCATTGGTCACAAATTGACTGTTAC	MYB	-	[0.12]
	89	TTGGTCACAAATTGACTGTTACAAC	MYB	-	[0.26]
	90	TCACAAATTGACTGTTACAACACCA	MYB	-	[0.37]
	91	AATTGACTGTTACAACACCATTTCA	MYB	4602.1	0.85
	92	GTTACAACACCATTTCATAGAGACC	MYB	4602.1	0.8
	93	ACAACACCATTTCATAGAGACCAGA	MYB	4602.1	0.83
	94	ACACCATTTCATAGAGACCAGACTG	MYB	4602.1	0.82
	95	CCATTTCATAGAGACCAGACTGTGA	MYB	4602.1	0.66
	96	CATAGAGACCAGACTGTGAAAACTC	MYB	-	[0.5]
1474_s_at	97	AGATGCACCGAATATTCTTACAAGC	MYB	4602.1	0.89
	98	GCACCAGCATCAGAAGATGAAGACA	MYB	-	[0.53]
	99	AGACAATGTTCTCAAAGCATTTACA	MYB	4602.1	0.72
	100	ATGTTCTCAAAGCATTTACAGTACC	MYB	4602.1	0.8
	101	TCTCAAAGCATTTACAGTACCTAAA	MYB	4602.1	0.8
	102	TACAGTACCTAAAAACAGGTCCCTG	MYB	-	[0.21]
	103	GAGCCCCTTGCAGCCTTGTAGCAGT	MYB	-	[0.53]
	104	CCCTTGCAGCCTTGTAGCAGTACCT	MYB	4602.1	0.75
	105	GCAGCCTTGTAGCAGTACCTGGGAA	MYB	4602.1	0.76
	106	AGTACCTGGGAACCTGCATCCTGTG	MYB	4602.1	0.77
	107	GAACCTGCATCCTGTGGAAAGATGG	MYB	4602.1	0.74
	108	AGCAGATGACATCTTCCAGTCAAGC	MYB	-	0.58*
	109	GATGACATCTTCCAGTCAAGCTCGT	MYB	4602.1	0.82
	110	CATCTTCCAGTCAAGCTCGTAAATA	MYB	4602.1	0.84
	111	TTCCAGTCAAGCTCGTAAATACGTG	MYB	4602.1	0.84
	112	GTCAAGCTCGTAAATACGTGAATGC	MYB	4602.1	0.84

probe_set	Probe #	sequence	Remap	Cleaner	consistency
	113	CCACACCAGACCTCATGGAGACAGT	MYB	4602.1	0.67
	114	GACCTCATGGAGACAGTGCACCTGT	MYB	4602.1	0.69
	115	GGAGACAGTGCACCTGTTTCCTGTT	MYB	-	[0.28]
	116	GTGCACCTGTTTCCTGTTTGGGAGA	MYB	-	[0.44]
	117	GGGAGAACACCACTCCACTCCATCT	MYB	-	[0.5]
	118	TCCTGGCTCCCTACCTGAAGAAAGC	MYB	-	[0.52]
	119	TCCCTACCTGAAGAAAGCGCCTCGC	MYB	-	[0.37]
1475_s_at	120	CGCCTCGCCAGCAAGGTGCATGATC	MYB	4602.1	0.66
	121	GCCAGCAAGGTGCATGATCGTCCAC	MYB	4602.1	0.79
	122	GTGCATGATCGTCCACCAGGGCACC	MYB	4602.1	0.77
	123	GATCGTCCACCAGGGCACCATTCTG	MYB	4602.1	0.71
	124	CACCAGGGCACCATTCTGGATAATG	MYB	4602.1	0.67
	125	GGCACCATTCTGGATAATGTTAAGA	MYB	4602.1	0.69
	126	TAAGAACCTCTTAGAATTTGCAGAA	MYB	-	[0.49]
	127	AGAACCACACATGCAGCTACCCCGG	MYB	-	[0.49]
	128	CCCGGGTGGCACAGCACCACCATTG	MYB	-	[0.44]
	129	AGCTATCAAAAGGTCAATCTTAGAA	MYB	-	[0.22]
	130	CAATCTTAGAAAGCTCTCCAAGAAC	MYB	-	[0.48]
	131	GAAAGCTCTCCAAGAACTCCTACAC	MYB	4602.1	0.7
	132	TCCAAGAACTCCTACACCATTCAAA	MYB	-	[0.36]
	133	CATTCAAACATGCACTTGCAGCTCA	MYB	4602.1	0.87
	134	TGCACTTGCAGCTCAAGAAATTAAA	MYB	-	0.58*
	135	TACGGTCCCCTGAAGATGCTACCTC	MYB	4602.1	0.64
1476_s_at	136	TGAAGATGCTACCTCAGACACCCTC	MYB	4602.1	0.57
	137	ACACCCTCTCATCTAGTAGAAGATC	MYB	4602.1	0.68
	138	ATGGACCACCCTTACTGAAGAAAAT	MYB	4602.1	0.7
	139	GAGGTGGAATCTCCAACTGATAAAT	MYB	4602.1	0.74
	140	GAATCTCCAACTGATAAATCAGGAA	MYB	-	[0.4]
	141	AAATCAGGAAACTTCTTCTGCTCAC	MYB	4602.1	0.86
	142	AACTTCTTCTGCTCACACCACTGGG	MYB	4602.1	0.61
	143	TGAATACCCAACTGTTCACGCAGAC	MYB	4602.1	0.89
	144	ACTGTTCACGCAGACCTCGCCTGTG	MYB	-	[0.53]
	145	ACGTTTTTTGCTGCTATGGTCTTAG	MYB	4602.1	0.89
	146	TTTTGCTGCTATGGTCTTAGCCTGT	MYB	4602.1	0.9
	147	TGCTGCTATGGTCTTAGCCTGTAGA	MYB	4602.1	0.89
	148	GTCTTAGCCTGTAGACATGCTGCTA	MYB	4602.1	0.9
	149	GCCTGTAGACATGCTGCTAGTATCA	MYB	4602.1	0.91
	150	GTAGACATGCTGCTAGTATCAGAGG	MYB	4602.1	0.89
	151	ACATGCTGCTAGTATCAGAGGGGCA	MYB	-	[0.53]
2042_s_at	152	TGCTGCTAGTATCAGAGGGGCAGTA	MYB	-	[0.39]
	153	TATCAGAGGGGCAGTAGAGCTTGGA	MYB	-	[0.53]

probe_set	Probe #	sequence	Remap	Cleaner	consistency
	154	TGACTATGCACTAGTATTTCAGACT	MYB	4602.1	0.88
	155	TATGCACTAGTATTTCAGACTTTTT	MYB	4602.1	0.86
	156	GCACTAGTATTTCAGACTTTTTAAT	MYB	4602.1	0.84
	157	TTCCTTCTGCAATACATTTGAAAAC	MYB	4602.1	0.85
	158	TTCTGCAATACATTTGAAAACTTGT	MYB	-	[0.22]
	159	TTTGAAAACTTGTTTGGGAGACTCT	MYB	-	[0.22]
	160	ACTTGTTTGGGAGACTCTGCATTTT	MYB	4602.1	0.74
41854_at	161	TTTTCTTCAGAAGGACTATAATCAG	-	-	-
	162	AAGACCCTGAGAAGGAAAAGCGAAT	MYB	-	[0.04]
	163	GAATTAGAATTGCTCCTAATGTCAA	MYB	-	[0.43]
	164	AGGTGCTACCAGTAAGACTGTCATC	-	-	-
	165	TACCAGTAAGACTGTCATCATGTGC	-	-	-
	166	CTGTCATCATGTGCTTGAATGAGGG	-	-	-
	167	GGGATAGCAGCTTTGCCTCAGTTTA	-	-	-
	168	AGCAGCTTTGCCTCAGTTTACCTAA	-	-	-
	169	TGCCTCAGTTTACCTAAGCGCTCTT	-	-	-
	170	GCCTCAGTTTACCTAAGCGCTCTTC	-	-	-
	171	AGCAAGGCTCCATATATCCATTCAG	-	-	-
	172	AGGCTCCATATATCCATTCAGAATG	-	-	-
	173	ATTCAGAATGTCTCAACACAAGAAG	-	-	-
	174	CAGAATGTCTCAACACAAGAAGTTG	-	-	-
	175	GTCTCAACACAAGAAGTTGCTTGTA	-	-	-
	176	CAACACAAGAAGTTGCTTGTAGTAA	-	-	-
